# Intra-Abdominal Hypertension Causes Bacterial Growth in Lungs: An Animal Study

**DOI:** 10.1155/2017/4601348

**Published:** 2017-03-05

**Authors:** Eleni Papakrivou, Demosthenes Makris, Efstratios Manoulakas, Magda Mitroudi, Konstantinos Tepetes, Konstantinos Papazoglou, Epaminondas Zakynthinos

**Affiliations:** ^1^Department of Critical Care Medicine, University Hospital of Larissa, University of Thessaly School of Medicine, Larisa, Greece; ^2^Department of Pediatric Surgery, General Hospital of “G. Gennimatas”, A' University Department, Aristotle University of Thessaloniki, Thessaloniki, Greece; ^3^Department of General Surgery, University Hospital of Larisa, University of Thessaly School of Medicine, Larisa, Greece; ^4^Department of Pathologo-Anatomy, General Hospital of “G. Gennimatas”, Thessaloniki, Greece

## Abstract

To study the effect of intra-abdominal hypertension (IAH) on the frequency of pneumonia with an experimental study, thirteen Sprague-Dawley rats were included. Eight out of thirteen animals were randomly assigned to receive 10 ml of benzalkonium chloride 0.2% (megacolon group) and five animals received 10 ml NaCl 0.9% (controls). Animals were anaesthetized by intramuscular delivery of ketamine. The incidence of positivity for bacteria lung tissue cultures and mesenteric lymph node cultures was assessed at the 21st day after animals' sacrification, or before in case of death. All megacolon group animals presented progressive increase of the abdomen and increased IAP (≥10 mmHg) whereas the frequency of their evacuations was almost eliminated. Controls presented normal evacuations, no sign of abdominal distention, and normal IAP. In megacolon group animals, there was evidence of significant amount of bacteria in lung cultures. In contrast, no bacteria were found in control animals.

## 1. Introduction

Animal experimental and human physiologic studies suggested that intra-abdominal hypertension (IAH) can cause impairment of respiratory function [1–3]. However, it is unknown whether IAH is associated with pneumonia [4]. This may have great importance for critical care patients who present IAH during their stay in the intensive care unit where pneumonia is a factor for increased morbidity and mortality [5].

Previous experimental data [6] have shown that IAP induced by megacolon is associated with loss of intestinal barrier and bacterial translocation to spleen, liver, and mesenteric lymph nodes. However, data regarding IAP and pneumonia are limited [4]. We conducted an experimental study by using an animal model to investigate whether an increased intra-abdominal pressure causes bacterial growth in mesenteric lymph nodes and lungs. This could support the hypothesis that translocation of bacteria from the abdominal cavity to the lungs could be also a responsible mechanism for pneumonia in IAH.

## 2. Materials and Methods

Thirteen Sprague-Dawley rats (weighted between 255 and 300 gr) were included in the experiment. Eight out of thirteen animals were randomly assigned to receive 10 ml of benzalkonium chloride 0.2% (megacolon group) and five animals received 10 ml NaCl 0.9% (controls), injected both under the serosal tissue, 1 cm from intrinsic sphincter of the rectum for 15 min. Animals were anaesthetized by intramuscular delivery of ketamine 50 mg/kg body-weight. They had then free access to water and laboratory rat chow and their height, weight, and perimeter of the abdomen and frequency of evacuations were monitored. The incidence of positive for bacteria lung tissue cultures and mesenteric lymph node cultures was assessed at the 21st day after animals' sacrification, or before in case of death. We further assessed histologically lung and colon specimens and lymph nodes.

### 2.1. Intra-Abdominal Pressure Measurement

Intra-abdominal pressure (IAP) was evaluated at 24 hours and at 21 days following benzalkonium chloride infusion (when animals were sacrificed) or before the 21st day if animals were moribund or presented clinical evidence of megacolon. Benzalkonium chloride infusion day was counted as Day Zero. All animals were examined clinically every day at 8:30 a.m. and then IAP measurements were performed at 9.00 a.m. In all cases each measurement was repeated twice and if the difference between measurements was >1 cmH_2_O, measurements were repeated. The mean measurement was reported. IAP was measured by a 16 F catheter positioned in the peritoneal cavity under sterile conditions and then connected to a mercury pressure system that included a mercury column, an elastic tube, and a three-way stopcock. The catheter entered directly into the abdomen taking care not to aspirate any intestinal context because of accidental intestinal penetration. The zero-reference was at the symphysis pubis [7].

The experimental protocol was approved by local Ethics Committee (Experimental Animal Ethics Branch-Approval No. EL 54 BIO 32, No. ADA:VIKZ7LL-G61, Protocol No: 105947/991) and under veterinarian supervision (TS). All animals were cared for and all procedures followed were in accordance with the ethical standards (institutional and national) and with Helsinki Declaration for the use and care of animals.

### 2.2. Histology and Microbiology

To obtain postmortem samples, we operated the abdominal cavity via a median umbilical incision under sterile conditions in the experimental operating room. A part of large intestine was sent for histological analysis, for assessment of the intestinal wall and samples of small bowel and inferior lung lobes were removed for histopathological examination. The tissue specimens were fixed in 10% formaldehyde, then dehydrated, and embedded in paraffin wax. The samples were sectioned and stained with hematoxylin and eosin (H&E) and assessed in a blinded fashion by one pathologist. As far as the histologic sampling and processing of lung tissues we followed a methodology [8], in which after the excision of each lung from the thoracic cage, the lungs were exposed on a hard surface. All lobes of both lungs were evaluated macroscopically (color, texture) by one pathologist. When macroscopic abnormalities, that is, foci of condensation (white-yellowish, imprecisely circumscribed, centered by bronchiole, and separated by normal lung parenchyma), could be identified, three specimens (1-2 mm, 0.5 gr) were sampled from the region of interest. In the case where macroscopic assessment of the lungs was normal, three specimens (1-2 mm, 0.5 gr) from the lower lobe of each lung were sampled (anterior, posterior, and lateral basal segment). Tissues were fixed with neutral formalin 10%, embedded in paraffin, and then manually sectioned with a microtome to obtain 4-5 *μ*m thick paraffin sections. Dewaxed sections were then stained with hematoxylin and eosin (H&E).

Liver, spleen, kidneys, bowel, ileac mesenteric lymph nodes, and lung tissue from the inferior lung lobes were examined microbiologically. The bacterial count was calculated as the log of colony-forming units (CFUs) per gram of tissue. All sampled tissues were weighed and homogenized separately in sterile plastic bags (Stomacker, Lab-Blender 80) with Teflon-coated tissue-grinding robs in phosphate-buffered saline. Each homogenate was diluted 1 : 3 and then plated on blood and MacConkey's agar culture plate. The plates were examined 24 and 48 hrs after incubation at 37°C [9].

A grading system of five grades of severity was used for histologic evaluation of the intestine [10]: Grade I: normal histology, Grade II: serosa oedema, hyperaemia, petechial hemorrhage, and early inflammation, Grade III: extensive epithelial separation from the lamina propria down the sides of the villi and ulcerations at the villus tips, Grade IV: full thickness, gangrene, and pneumatosis, and Grade V: perforation. Moreover, in order to classify histologic results of animals' lungs we used a classification of severity of lung infection based on the classical description of pneumonia stages [11] where + means normal, ++ means congestion, +++ means consolidation, ++++ means grey hepatization, and +++++ means resolution.

### 2.3. Statistical Analysis

Data are expressed as mean (SD). Data were compared using Fisher's exact test for categorical variables or the *t*-test or the Mann–Whitney* U* test for continuous variables as appropriate. All statistical tests were 2-sided. A result was considered statistically significant when *p* < 0.05. Analysis was performed using SPSS 11.0 for Windows.

## 3. Results

There was no difference between megacolon group and control animals in terms of baseline characteristics (age, weight, height, IAP, and evacuations/day). Controls presented normal evacuations, no sign of abdominal distention, and normal IAP. No animal died during the 21-day period (Table 1). Megacolon group animals presented progressive increase of the abdomen perimeter (*p* = 0.001), higher IAP (≥10 mmHg) (*p* = 0.0001), and decreased frequency of their evacuations (*p* = 0.001) compared to control animals between the first day and the 21st day of assessment.

**Figure 1:**
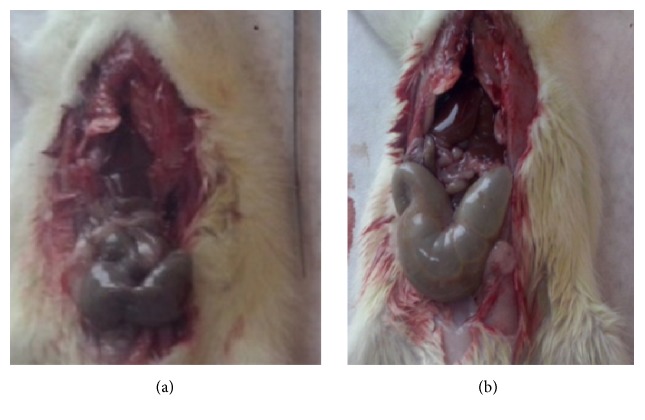
(a) Normal diameter of the intestine in control group. (b) In MG animals we observed that the diameter of the tube of small intestine was minimized and the large bowel had a significant dilatation.

**Figure 2:**
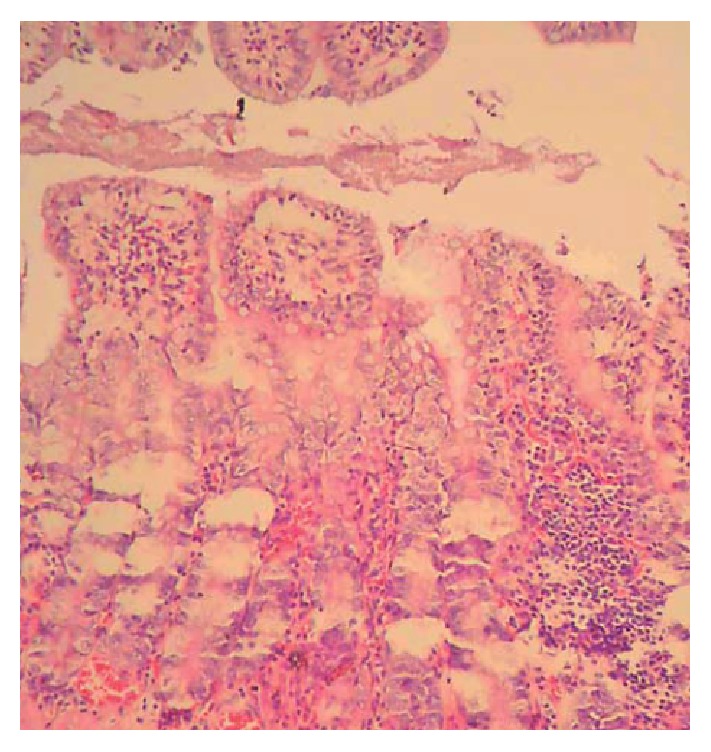
Histological findings of megacolon group: extensive epithelial separation from the lamina propria down the sides of the villi, ulceration at the villus tips, and severe inflammation extending deep through all the layers of the colon with infiltration by neutrophils, lymphocytes, histiocytes, and plasma cells (H&E, 40).

At autopsy the diameter of the intestine in control group was normal (Figure 1(a)). In megacolon group animals we observed that the diameter of the tube of small intestine was minimized and the large bowel had a significant dilatation (Figure 1(b)) and the wall of intestine presented ischemic ulcerations. Histological analysis showed extensive epithelial separation from the lamina propria down the sides of the villi, ulceration at the villus tips, and mucosal injuries of Grades III and IV (Figure 2).

**Figure 3:**
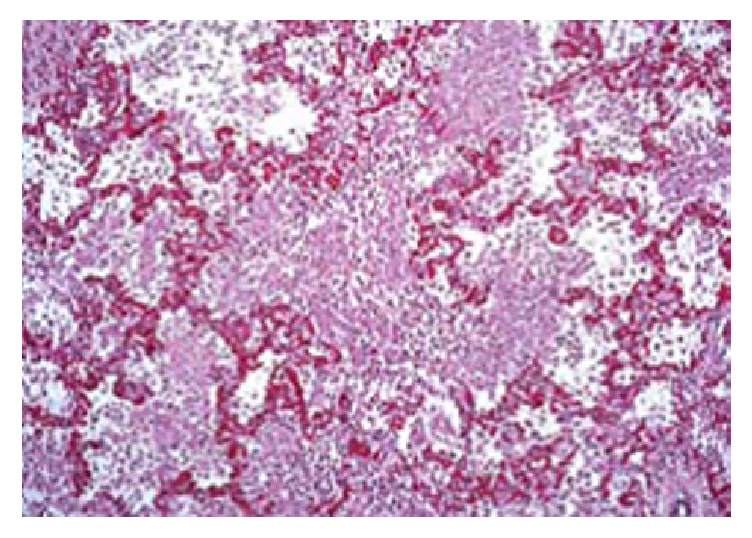
Histological picture of the lung of an animal with intra-abdominal hypertension following megacolon, revealing alveolar septal dilatation and edema and inflammatory infiltrates of mononuclear lymphocytes, histiocytes, plasma cells, and neutrophils.

**Figure 4:**
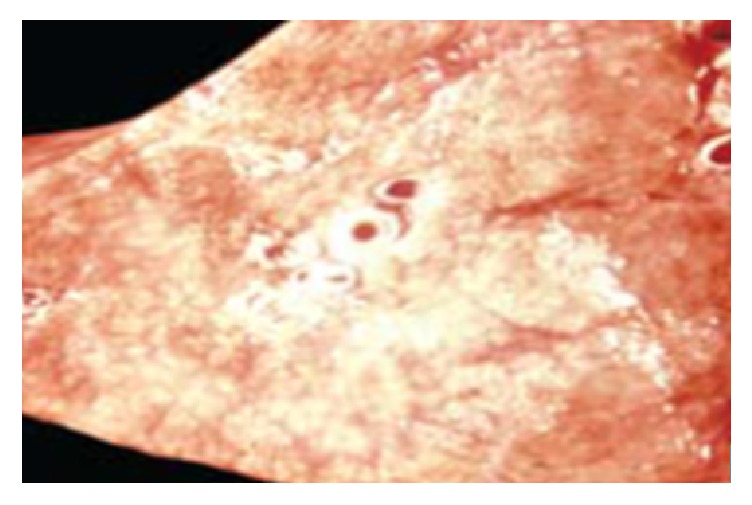
Gross appearance of solidification and consolidation of alveolar parenchyma.

**Figure 5:**
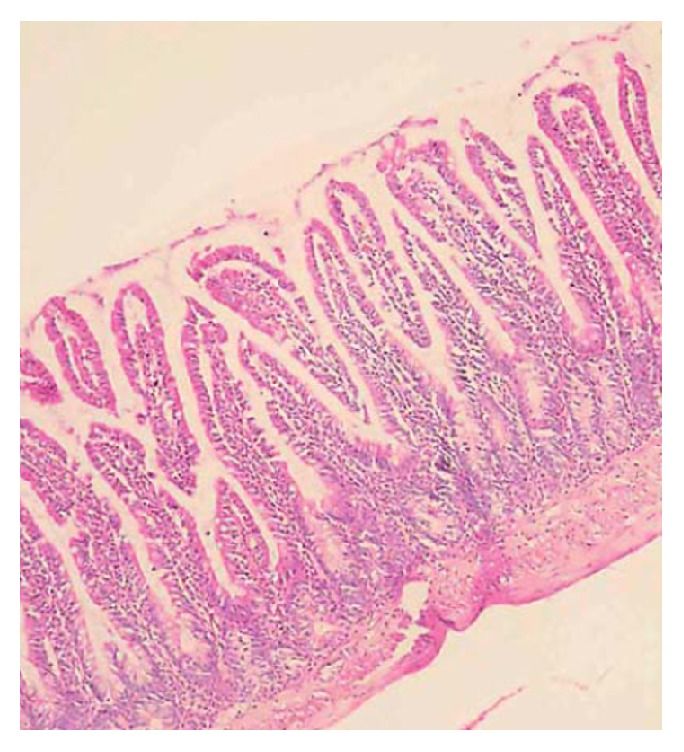
Animal in control group without signs of severe inflammation and with the presence of all intestinal layers without necrosis (H&E, 40).

Histological examination of the lung tissue revealed edema of the alveolar septal and infiltration by neutrophils, mononuclear lymphocytes, histiocytes, and plasma cells (Figure 3). Also, pathologoanatomical examination showed gross appearance of solidification and consolidation of alveolar parenchyma (Figure 4). In contrast, no histologic evidence of megacolon or inflammatory changes in the intestine or lungs were found in control animals (Figure 5). A histological classification of results is shown in Table 2.

In megacolon group animals, there was evidence of significant amount of bacteria in lung cultures; the most common bacteria were* Klebsiella* species (*pneumoniae* and* oxytoca*)* E. coli*,* Enterococcus faecalis*,* Peptostreptococcus (anaerobius*,* magnus)*, and* Pseudomonas aeruginosa. *Bacteria were also found in the mesenteric lymph nodes, spleen, liver, and kidneys. Isolated bacteria in mesenteric lymph nodes and lungs were the same in all animals (Table 3). In contrast, no bacteria were found in control animals.

## 4. Discussion

The present study demonstrated that an increase in IAP in studied animals, following chemical megacolon, was associated with lung inflammatory changes and similar bacterial growth in the lungs and in mesenteric lymph nodes. These phenomena were not observed in control animals. These findings may indicate that the responsible underlying mechanism for pneumonia in cases where intra-abdominal pressure is highly elevated could be bacterial translocation via the bloodstream, besides the aspiration of gastrointestinal content which is a known pathophysiologic mechanism.

Previous studies reported that when intra-abdominal pressure increases, bacterial translocation to mesenteric lymph nodes may occur. This was reported with the presence of an IAP of 10 mmHg [11]. In our study the mean IAP at rats was 13.8 mmHg and bacterial translocation may be suggested by the detection of several bacteria (i.e.,* E. coli*,* Enterococcus faecalis*,* Pseudomonas aeruginosa*, and* Peptostreptococcus*) in regional mesenteric lymph nodes but also in regional and distal organs such as the lungs of the animals.

Both major trauma or abdominal injury may lead to the development of increased intra-abdominal pressure with adverse effects on systemic and splanchnic hemodynamics [12]. Data on the consequences of the resultant gut hypoperfusion in this setting are very limited. However, it is clear that bacterial translocation may occur after a period of splanchnic ischemia in the setting of IAH. Liu et al. [11] used a rodent model to examine the effect of raised IAP on ileal mucosal blood flow and on bacterial translocation. An increased IAP of 25 mmHg for 60 minutes resulted in significantly decreased mucosal blood flow (−63% from baseline) in rodents despite the maintenance of normal mean arterial blood pressure.

In Diebel et al. study, bacterial translocation occurred principally to the mesenteric lymph nodes [13]. The latter requires the passage of bacteria from the lumen to the submucosa. Cells containing immunoreactive material are generally more concentrated around vascular structures in the submucosa, where invading pathogens would have ready access to the rich lymphatic plexus, thereby reaching local lymph nodes [14]. When bacteria escape the immunologic mechanisms of the mesenteric lymph nodes they can reach the efferent lymphatic vessels and, through the thoracic duct, can enter the systemic circulation and translocate to distal organs. It is noteworthy to underline that IAH can be the reason for this augmented TNF production in the MLN of cirrhotic rats [15] and cause bacterial translocation from the intestinal lumen to the MLN. Bacterial antigens and endotoxins are potent stimulators of TNF release by mononuclear cells. This relationship between TNF production and bacterial translocation was supported by Genescà et al. [15], by the observed association of high TNF levels in MLN from rats with advanced liver failure and more importantly with the presence of ascites and IAH, as both factors seem to correlate with bacterial translocation. Cirrhotic rats need to develop ascites in order to present efficient bacterial translocation, and when bacteria translocate in MLN, local TNF production is substantially increased. It should be also noted that the mesenteric lymphatic drainage seems to play a role as a path modulator of the pulmonary and intestinal dysfunctions. This has been demonstrated in both gut trauma [16] and hemorrhagic shock-induced lung injury [17] whereas mesenteric lymphatic ducts ligation can decrease the degree of gut-induced lung injury [11].

The relationship that was found in our study between IAP and pneumonia in megacolon group animals suggests a link between them. As explained previously, we speculate that bacterial translocation might be a responsible mechanism for that relationship beside gastric aspiration. Yet, it is difficult to prove direct causality between them and our study cannot clarify whether, and to which extend, bacterial translocation may be via the bloodstream or the gastric lumen. The answer to such hypothesis would probably require, in addition, the assessment of inflammatory colonic parameters (such as cyclooxygenase-2, inducible nitric oxide synthase, and myeloperoxidase), colonic immunoglobulin A [18], or the evaluation of levels of gastric enzymes in the lung, that is, pepsin [19]. A study is under way to test this hypothesis.

In the present study we used a model of IAP based on chemical megacolon development following intestinal application of benzalkonium chloride in rats which has been used in other studies in the past [20]. The application of benzalkonium chloride may serve as an animal model of congenital megacolon due to the adverse effects of benzalkonium chloride on myenteric neurons after acute (until 10 days after its application) or chronic (30 and 60 days after application) denervation of the proximal jejunum [21]. In our study we observed megacolon in all treated animals and that IAP was significantly increased in those animals at the 21st day assessment. IAH could reasonably attribute to megacolon firstly, because megacolon is a known cause of IAH [22] and, secondly, because there was no evidence of other known insult that could cause IAH (i.e., major abdominal trauma and abdominal ischemia).

In contrast to chemical megacolon, a different method of IAP induction (i.e., a septic abdomen) might have been closer to conditions that may be encountered in clinical practice (i.e., IAH and pneumonia are usually seen in critical care patients with abdominal sepsis). However, our main question was the relationship between IAP and pneumonia and in this respect; it is well known that megacolon can induce IAH [22]. Preliminary experiments in our setting (not shown) have tested this hypothesis and we therefore used the current model in our study. In addition, chemical megacolon causes gradual increase of intra-abdominal pressure allowing a sufficient time period for development of pneumonia [22] whereas megacolon models have been used in the literature to evaluate translocation in local and distal lymph nodes [23].

In the present model, we used a direct method of IAP measurement that included the insertion of a thin catheter (16 F) into the abdominal cavity. We followed this method of two measurements because we considered it as a relative less invasive process compared to others [24], in order to minimize the risk of intestine perforation/hemorrhagic complications or infection and thus to avoid secondary insults that may have obscured our results. Moreover, this method of measurement does not need prolonged anesthesia, maintenance of catheters in the abdominal cavity, or frequent manipulations which could increase per se the risk of bacterial dispersion in the abdomen.

We certainly acknowledge that some other points should be taken into consideration when interpreting our results. Despite the fact that animal models of intra-abdominal hypertension are indispensable tools in research, model-related drawbacks may interfere with one or more pathophysiological aspects of our investigation; that is, rodents may present bacterial translocation more often than humans [25]. In addition, all lobes were examined macroscopically for abnormalities and all regions of interest or at least three specimens from the lower lobe of each lung were sampled but we have not assessed all lobes microscopically; in this respect an area with minimal inflammation may have been missed. However, in previous studies a similar methodology was followed [26]. Finally, we used clinical (weight, height, abdominal perimeter, and frequency of evacuations), autopsies, and histologic criteria for the assessment of megacolon but radiologic evaluation was not used. However, we believe that this has not decreased the accuracy of megacolon diagnosis in treated animals since all of them proved to have megacolon based on autopsy-histology.

## 5. Conclusions

In conclusion, the present investigation suggests that IAH may be a risk factor for pneumonia and that bacterial translocation might be an additional responsible mechanism for pneumonia which merits further investigation in the future.

## Figures and Tables

**Table 1:** Characteristics of animals participated in the study (megacolon group numbers 1–8, control group numbers 9–13).

Animal number	Height (cm)		Weight (gr)		Abdominal perimeter (cm)		Evacuations per day (*n*)		IAP	
	24 hours	21 days	24 hours	21 days	24 hours	21 days	24 hours	21 days	24 hours	21 days
1	18.2	18.3	256.7	261.1	16.2	18.2	3	1	13	18
2	17.4	17.4	255.3	262.2	17.3	20.4	5	0	14	19
3	18.6	19.2	270.3	289.4	18.1	22.3	2	2	14	19
4	19.5	19.5	267.4	300.5	16.2	17.1	4	0	15	20
5	16.3	17.3	278.3	292.2	17.2	18.1	3	0	15	20
6	18.1	18.1	256.3	265.1	16.4	17.3	4	1	15	18
7	18.2	18.1	275.3	298.1	16.3	17.5	4	0	13	18
8	18.3	19.2	255.5	265.5	16.6	17.6	5	1	13	19

Mean (SD)	18.1 (0.9)	18.4 (0.8)	264.4 (9.6)	279.3 (17.3)	16.8 (0.7)	18.6 (1.8)^*∗*^	3.8 (1)	0.6 (0.7)^*∗*^	14 (0.9)	18.9 (0.8)^*∗*^

9	18.4	18.5	257.6	260.1	16.1	16.2	3	4	8	9
10	17.1	17.2	286.7	288.1	17.5	17.5	4	3	9	7
11	18.1	18.1	287.1	288.3	15.4	15.6	4	4	8	8
12	17.1	18.1	267.1	270.1	16.3	16.4	3	3	8	9
13	18.1	19.3	270.3	273.5	17.1	17.1	3	3	7	7

Mean (SD)	17.8 (0.6)	18.2 (0.8)	273.8 (13)	276 (12.2)	16.5 (0.8)	16.6 (0.8)	3.4 (0.6)	3.4 (0.6)	8 (0.7)	8 (1)

IAP = intra-abdominal pressure.

^*∗*^ *p* < 0.05 for the difference between 24 hours and 21 days.

**Table 2:** Histological results of megacolon group and control group (megacolon group numbers 1–8, control group numbers 9–13).

Animal number	Severity/stage of pneumonia
1	++
2	+++
3	++
4	++++
5	++++
6	+++
7	+++
8	++
9	+
10	+
11	+
12	+
13	+

+ = *normal*, ++ = *congestion*, +++ = *consolidation*, ++++ = *grey hepatization*, and +++++ = *resolution*.

**Table 3:** Type and number of viable isolated bacteria (mean CFUs/g of tissue) in animals with increasing IAP (mmHg) after chemical megacolon (animals 1–8) and controls (animals 9–13).

Animal number	MLN	Spleen	Liver	Kidney	Lung
1	*Klebsiella pneumoniae*	*Enterococcus faecalis*	*Enterococcus faecalis*	*E. coli*	*Klebsiella pneumoniae*
	33 × 10^3^	18 × 10^3^	31 × 10^3^	14 × 10^3^	20 × 10^3^
2	*Klebsiella pneumoniae*	*E. coli*	*E. coli*	*Klebsiella pneumoniae*	*Klebsiella pneumoniae*
	35 × 10^3^	19 × 10^3^	33 × 10^3^	15 × 10^3^	21 × 10^3^
3	*Enterococcus faecalis*	*Proteus mirabilis*	Peptostreptococcus	*P. aeruginosa*	*Enterococcus faecalis*
	36 × 10^3^	19 × 10^3^	33 × 10^3^	15 × 10^3^	21 × 10^3^
4	*P. aeruginosa*	Peptostreptococcus	Peptostreptococcus	Peptostreptococcus	*P. aeruginosa*
	38 × 10^3^	21 × 10^3^	34 × 10^3^	17 × 10^3^	23 × 10^3^
5	*P. aeruginosa*	*Proteus mirabilis*	Peptostreptococcus	*Proteus mirabilis*	*P. aeruginosa*
	38 × 10^3^	21 × 10^3^	34 × 10^3^	16 × 10^3^	23 × 10^3^
6	*E. coli*	*P. aeruginosa*	*P. aeruginosa*	*P. aeruginosa*	*E. coli*
	32 × 10^3^	18 × 10^3^	31 × 10^3^	14 × 10^3^	19 × 10^3^
7	*Klebsiella oxytoca*	Peptostreptococcus	*Proteus mirabilis*	*Proteus mirabilis*	*Klebsiella oxytoca*
	31 × 10^3^	19 × 10^3^	31 × 10^3^	14 × 10^3^	19 × 10^3^
8	*Klebsiella pneumoniae*	*P. aeruginosa*	*P. aeruginosa*	*Proteus mirabilis*	*Klebsiella pneumoniae*
	35 × 10^3^	19 × 10^3^	32 × 10^3^	15 × 10^3^	20 × 10^3^
9	—	—	—	—	—
10	—	—	—	—	—
11	—	—	—	—	—
12	—	—	—	—	—
13	—	—	—	—	—

IAP = intra-abdominal pressure; MLN = mesenteric lymph nodes of ileus.

## Abbreviations

CFUs: Colony-forming units
IAH: Intra-abdominal hypertension
IAP: Intra-abdominal pressure
MLN: Mesenteric lymph nodes.
